# 

**Published:** 1859-01

**Authors:** 


### RECEIPTS FOR JOURNAL UP TO FEBRUARY 1st, 1859.

### Illinois.

| | | |
|---|---|---|
| Dr. L D. Smedley, | vol. 2, 1859, | $2 00 |
| " J. L. Hoover, | " " | 2.00 |
| " H. D. Adams, | " " | 2 00 |
| " E L. Holmes, | " " | 2.00 |
| " Isaac Price, | " " | 2.00 |
| " Samuel M. Mitchell, | " " | 2.00 |
| " A. W. Butler, | " " | 2.00 |
| " L. W. Carter, | " " | 1.00 |
| " J. M. C. Damron, | " " | 2.00 |
| " W. H. Lyford, | " " | 2.00 |
| " L. B. Brown, | " " | 2.00 |
| " W. S. H. Birney, | " " | 2.00 |
| " D. O B. Adams, | " " | 2.00 |
| " B. F. Ross, | " " | 2.00 |
| " D. W. Stormont, | " " | 2 00 |
| " J. M. Steele, | " " | 2.00 |
| " Samuel McNair, | " " | 2.00 |
| " George Vesey, | " " | 2.00 |
| " D. D. Thompson. | " 1, 1858, | 2.00 |
| " A. Marshall Miller. | " " | 2.00 |
| " R. F. Henry, | " " | 2 00 |
| " S. York, | " " | 2 00 |
| " Owen Wright, | " " | 2.00 |
| " W. W. Walker. | " " | 2.00 |
| " John Bowman, | " " | 1 00 |
| " F. Ehrhardt, | " " | 2.00 |
| " J. A Hill, | vols. 1 & 2. 1858-9, | 5.00 |
| " B. Woodward, | " 6, 1, 2. 1857-8-9, | 5.00 |
| " J. H. Frizell, | " 6 & 1, 1857-8, | 4.00 |
| " J Grinstead, | " " " | 4.20 |
| " S. Kelly, | vols. 5, 6 & 1. 1856-7-8, | 6.00 |
| " J. P. Ross, | " 4. 5 & 2. 1855-6-9, | 6.00 |
| "Esculapian Society," | | 10.00 |

### California

| | | |
|---|---|---|
| Dr. J. R. Bradway, | vol. 1, 1858, | $2.50 |

### Iowa.

| | | |
|---|---|---|
| Dr. J. W. Cowden, | vol. 2, 1859, | $2 00 |
| " A. A. & H. C. Rawson, | " " | 2. 0 |
| " Van Nus, | " 1, 1858, | 2.00 |

### Wisconsin.

| | | |
|---|---|---|
| Dr. A. P. Adams, | vol. 2. 1859, | $2 00 |
| " M. Waterhouse. | " " | 1.00 |
| " C. Marsh, | " 1, 1858, | 2.00 |

### Indiana.

| | | |
|---|---|---|
| Dr. R. W. Pierce, | vol. 2. 1859, | $2.00 |
| " M. H. Bonnell, | " " | 2.00 |
| " D. B. Montgomery, | " " | 2.00 |
| " F Baird, | " " | 2.00 |
| " Laughlin O'Neil, | " " | 2.00 |
| " J. P. Graham, | " " | 2.00 |
| " A. W. Adair, | " " | 2.00 |
| " Hiram Duncan, | " 1, 1858, | 2 00 |
| " T Newkirk, | " " | 2 00 |
| " J. W. Green, | vols. 6, 1. 2, 1857-8-9, | 5.00 |
| " A Harrison, | " 4, 5, 6, 1, 1855-6-7-8, | 8.00 |

### Missouri.

| | | |
|---|---|---|
| Dr. C. M. Robertson, | vol. 1, 1858, | $2.00 |

### Kansas.

| | | |
|---|---|---|
| Dr. Wm Manson, | vol. 2. 1859, | $2.00 |

